# Mental health in the post COVID-19 era: future perspectives

**DOI:** 10.31744/einstein_journal/2022CE6760

**Published:** 2022-04-25

**Authors:** Ana Carla dos Santos Costa, Vaishnavi Menon, Rachana Phadke, Kartik Dapke, Adriana Viola Miranda, Shoaib Ahmad, Mohammad Yasir Essar, Hashim Talib Hashim

**Affiliations:** 1 Faculdade de Medicina Universidade Federal da Bahia Salvador BA Brazil Faculdade de Medicina, Universidade Federal da Bahia, Salvador, BA, Brazil.; 2 Indira Gandhi Government Medical College Nagpur MH India Indira Gandhi Government Medical College, Nagpur, MH, India.; 3 Faculty of Medicine University of Indonesia Jakarta DK Indonesia Faculty of Medicine, University of Indonesia, Jakarta, DK, Indonesia.; 4 Punjab Medical College Faisalabad PK Pakistan Punjab Medical College, Faisalabad, PK, Pakistan.; 5 Medical Research Center Kateb University Kabul AF Afghanistan Medical Research Center, Kateb University, Kabul, AF, Afghanistan.; 6 University of Baghdad College of Medicine Baghdah IQ Iraq University of Baghdad, College of Medicine, Baghdah, IQ, Iraq.

Dear Editor,

On March 11, 2020, the World Health Organization (WHO) characterized the coronavirus disease 2019 (COVID-19), which is caused by the severe acute respiratory syndrome coronavirus 2 (SARS-CoV-2), as a pandemic.^(1)^ The novel coronavirus disease has originated as a cluster of unexplained cases of pneumonia in Wuhan, China, and it has scaled to new heights of mortality by affecting people both physically and mentally.

The context of unpredictability and uncertainty associated with the lockdown, the numerous restrictions, unemployment, and changes in the standard of living, have limited the already precarious access to mental health services. In addition, these issues have been imposed a great psychological burden on the population. Consequently, an increase in the prevalence of mental disorders in previously healthy individuals were observed, as well as the aggravation of pre-existing mental disorders.^(2)^

In the general public, there was an increase in the incidence of depression, anxiety, stress, panic disorder, obsessive-compulsive disorder, somatic symptoms, sleep disorders, delirium, psychosis, self-mutilation, and suicide. In addition to the factors already mentioned, insecurity about the future, fear of contracting the disease, fear of the negative economic effects due to the pandemic, the lack of information and scientifically proven treatment are some of the factors responsible for these mental issues. The spread of false information, the scarcity of essential products, and fear also led to an increase in panic buying episodes. People with pre-existing mental disorders reported worsening symptoms, relapse, and suicidal behaviour due to the interruption of psychiatric care, home confinement, and changes in their daily routine. In addition, restrictive measures have reduced patients’ access to family and social support networks.^(2)^

In a study conducted in China, home confinement and centralized quarantine were found to be associated significantly with increased risk of psychological outcomes including anxiety, depression, insomnia, and acute stress.^(3)^ Furthermore, the lockdown measures have restricted people to their homes and infringed upon their freedom. This has resulted in various deleterious ways of coping with daily stressors, such as alcohol, drug and tobacco abuse, potentially addictive behaviours, such as online gaming and gambling, and rise in rates of domestic violence and sexual abuse.^(4)^

The stigma and fear associated with the disease have presented great barriers to seeking health care. Due to the lack of trust in the authorities, social marginalization, and distorted perception of risk by the public, there has been massive panic among citizens and disproportionate allocation of health resources by politicians and health professionals.^(5)^ These conditions have been exacerbated by the home confinement, disruption of daily routines, and physical distancing, in addition they represent a major challenge for service users and caregivers.

Among health professionals, the situation also was aggravated by exposure to daily stress, increased workload, burnout, discrimination, distance from the family, fear of becoming infected and transmitting the disease to close people, scarcity of resources, mainly personal protective equipment, and frequent contact with terminally ill patients due to the illness. In this context, the professionals most affected were those who were on the front lines fighting the pandemic.^(2)^ As an example, a study in China, which used Self-rating Anxiety Scale and Self-rating Depression Scale, showed that the median score of the frontline professionals on these two scales was significantly higher than those of the other members of the medical team, that is, frontline workers were more likely to present anxiety and depression.^(6)^

## Future perspectives

Given the ongoing global economic recession and long-term isolation, the deleterious impact of the pandemic to mental health is expected to not only to exist during the pandemic, but also in the post-pandemic era. Economic recession and social isolation are known to increase the rate of depression, anxiety, substance abuse disorders, and suicidal behaviour. Thus, it is expected that after the pandemic, the global health systems will experience increased demands for mental health care.^(2)^

Telepsychiatry provides an opportunity to effectively address the post-pandemic mental health needs. Telepsychiatry services with remote video or phone conferences have been effective to ensure adequate mental health care amidst the pandemic. Digital health applications that allow remote screening and patient monitoring are also being developed.^(7)^ Several advantages of Telepsychiatry for patients include increased access to care, reduction of travel and waiting time, and decreased cost. For clinicians, the major advantages of Telepsychiatry are scheduling flexibility, increased diversity of practices, and increased safety. However, concerns regarding the decreased ability to detect non-verbal cues and engagement with patients have been reported.^(8)^ Nevertheless, using Telepsychiatry in the post-pandemic era is still beneficial since it will enable mental health care to reach the wider public at an affordable cost and support the patient-psychiatrist interaction and monitoring in-between scheduled visits.

For healthcare professionals and frontline workers, it is essential that preventive interventions should be taken. It is important to educate them of the several behavioural strategies that can be followed for coping with stress. This can be done by implementing psychological intervention services that offer mental health support applications, regular monitoring for mental health illnesses, and provide early support. Also, it is important to conduct group discussions to help the staff members to openly talk about how the pandemic is affecting their work and to identify factors that cause stress and work together to identify solutions. In addition, an anonymous online self-check tool should be available for staff members at all institutions, to encourage honest and meaningful responses while providing automated tailored feedback.^(9)^

In the future, scientific research through longitudinal cohort study must be conducted to learn about the persistent psychological situation that is caused because of this looming crisis. More reliable research data should be used to formulate evidence-based prevention and treatment strategies, to reduce the adverse psychological impact caused by COVID-19 pandemic.

Enhancing the knowledge and confidence of primary health care physicians in treating these prevalent mental health disorders is one of the most effective ways to address this problem. All health care professionals should be encouraged to participate in special training courses on emergency psychological care and first aid as prepared by WHO and other organisations.

Additional training should also be provided to community health service professionals at the grass-roots level to help them master the methods to deal with psychological crisis, and actively communicate with the public to understand their mental state, this ensuring early detection and prevention of severe mental health issues. Online training may be used if it is not possible to train staff in person due to limited time.^(9)^

Every health care institute must have a system to identify and help provide care for patients with mental health conditions particularly during this time since the stigma associated with mental health can make people to avoid to seek for helping. It is essential to help patients acknowledge that mental health issues exist and that they need help to normalize it. Basic self-care strategies can be taught to patients to help them to recognize the signs of distress. Easy and simple ways to manage or control these signs must also be discussed. Encouraging people to prioritize their well-being, starting the discussion about mental health among company leaders, and educating people to consider their word choices surrounding mental health are simple steps that can be taken to overcome the stigma against mental health.

## CONCLUSION

The aftermath of coronavirus disease 2019 pandemic is likely to be a wave of psychiatric illnesses. These health issues stem from normal people being exposed to extraordinary situations. The presentations of symptoms can range from emotional difficulties like anxiety, depression or biological effects like sleep, appetite disturbances to severe mental illness. Increase in the prevalence of these disorders have resulted in an increased burden on the mental health care system across the world. Thus, some important measures that need to be taken to address this looming crisis are the early assessment and proper treatment as an attempt to improve the feasibility of access to support frontline work groups, the education of people about self-care strategies, the addressing of the stigma against mental health, and the building of additional services such as Telepsychiatry.
